# Noncommunicable Respiratory Disease and Air Pollution Exposure in Malawi (CAPS). A Cross-Sectional Study

**DOI:** 10.1164/rccm.201805-0936OC

**Published:** 2019-03-01

**Authors:** Rebecca Nightingale, Maia Lesosky, Graham Flitz, Sarah J. Rylance, Jamilah Meghji, Peter Burney, John Balmes, Kevin Mortimer

**Affiliations:** ^1^ Liverpool School of Tropical Medicine, Liverpool, United Kingdom; ^2^ Malawi Liverpool Wellcome Trust Programme, Blantyre, Malawi; ^3^ Division of Epidemiology and Biostatistics, School of Public Health and Family Medicine, University of Cape Town, Cape Town, South Africa; ^4^ University of California, Berkeley, California; ^5^ National Heart and Lung Institute, Imperial College, London, United Kingdom; and; ^6^ University of California, San Francisco, San Francisco, California

**Keywords:** household air pollution, Malawi, chronic obstructive pulmonary disease, Cooking and Pneumonia Study, cookstove

## Abstract

**Rationale:** Noncommunicable respiratory diseases and exposure to air pollution are thought to be important contributors to morbidity and mortality in sub-Saharan African adults.

**Objectives:** We set out to explore the prevalence and determinants of noncommunicable respiratory disease among adults living in Chikhwawa District, Malawi.

**Methods:** We performed a cross-sectional study among adults in communities participating in a randomized controlled trial of a cleaner-burning biomass-fueled cookstove intervention (CAPS [Cooking and Pneumonia Study]) in rural Malawi. We assessed chronic respiratory symptoms, spirometric abnormalities, and personal exposure to air pollution (particulate matter <2.5 μm in aerodynamic diameter [PM_2.5_] and carbon monoxide [CO]). Weighted prevalence estimates were calculated; multivariable and intention-to-treat analyses were done.

**Measurements and Main Results:** One thousand four hundred eighty-one participants (mean [SD] age, 43.8 [17.8] yr; 57% female) were recruited. The prevalence of chronic respiratory symptoms, spirometric obstruction, and restriction were 13.6% (95% confidence interval [CI], 11.9–15.4), 8.7% (95% CI, 7.0–10.7), and 34.8% (95% CI, 31.7–38.0), respectively. Median 48-hour personal PM_2.5_ and CO exposures were 71.0 μg/m^3^ (interquartile range [IQR], 44.6–119.2) and 1.23 ppm (IQR, 0.79–1.93), respectively. Chronic respiratory symptoms were associated with current/ex-smoking (odds ratio [OR], 1.59; 95% CI, 1.05–2.39), previous tuberculosis (OR, 2.50; 95% CI, 1.04–15.58), and CO exposure (OR, 1.46; 95% CI, 1.04–2.05). Exposure to PM_2.5_ was not associated with any demographic, clinical, or spirometric characteristics. There was no effect of the CAPS intervention on any of the secondary trial outcomes.

**Conclusions:** The burden of chronic respiratory symptoms, abnormal spirometry, and air pollution exposures in adults in rural Malawi is of considerable potential public health importance. We found little evidence that air pollution exposures were associated with chronic respiratory symptoms or spirometric abnormalities and no evidence that the CAPS intervention had effects on the secondary trial outcomes. More effective prevention and control strategies for noncommunicable respiratory disease in sub-Saharan Africa are needed.

Clinical trial registered with www.isrctn.com (ISRCTN 59448623).

**At a Glance Commentary**

**Scientific Knowledge on the Subject**

Noncommunicable respiratory diseases and exposure to air pollution are thought to be important causes of morbidity and mortality in sub-Saharan African adults. Recent BOLD (Burden of Obstructive Lung Disease) studies found a high burden of spirometric restriction but little spirometric obstruction in several sub-Saharan African countries and no association between spirometric obstruction and use of dirty-burning fuels. It is not known whether an association between spirometric obstruction and solid fuel use would be seen if personal exposure to air pollution were measured in addition to self-reported exposure. CAPS (Cooking and Pneumonia Study)—a trial of cleaner burning biomass-fueled cookstoves on pneumonia in children <5 years of age in rural Malawi—offered the opportunity to explore this and other secondary trial outcomes in adults.

**What This Study Adds to the Field**

In adults living in Chikhwawa, rural Malawi: 13.6% of participants had chronic respiratory symptoms (mainly cough); >40% had abnormal spirometry (mainly spirometric restriction); day-to-day air pollution exposures were approximately three times the World Health Organization upper safety limit; air pollution exposures were not associated with demographic, clinical, or spirometric characteristics; and there was no association between CAPS trial arm and any of the secondary trial outcomes.

Highly polluting fuels, including animal dung, crop residues, wood, charcoal, and kerosene, are used by almost half the world’s population to provide energy for cooking, heating, and lighting (1–3). These fuels are typically burned in and around the home environment in inefficient ways (e.g., open fires), which leads to high levels of air pollution in and immediately outside of homes. The World Health Organization (WHO) has estimated that exposure to household air pollution leads to >4 million deaths each year (3). The latest Global Burden of Disease Study estimates this number is closer to 2.5 million, but even these lower estimates represent a substantial burden of morbidity and mortality that falls particularly heavily on the world’s poor (4). Household air pollution has been considered to increase the risk of pneumonia in children and of chronic obstructive pulmonary disease (COPD) and cardiovascular disease in adults (1–3).

In 2017, we published the findings of a cluster-randomized controlled trial of introducing a cleaner-burning biomass-fueled cookstove to prevent pneumonia in children <5 years of age in rural Malawi (CAPS [Cooking and Pneumonia Study]) (5). CAPS is one of a small number of trials done to date to evaluate the effects of reducing biomass smoke exposure on health outcomes and is the largest trial of a cookstove intervention on health outcomes conducted anywhere in the world (*n* = 10,750 children from 8,626 households across 150 clusters). The major finding of this trial was that there was no difference between the intervention and control groups among children in pneumonia incidence defined using the criteria of the Integrated Management of Childhood Illness program. This unexpected finding has cast some doubt on the assumptions made by the Global Alliance for Clean Cookstoves that cleaner cookstoves and fuels save lives (6–11).

Herein we report the findings of a cross-sectional study of the prevalence and determinants of noncommunicable respiratory disease among adults living in communities that participated in CAPS, which addresses the prespecified secondary trial objective of determining prevalence and determinants of obstructive lung disease in adults in rural Malawi (5). In this setting, use of highly polluting fuels for day-to-day household energy requirements is the norm, and therefore a high burden of COPD associated with household air pollution was expected.

## Methods

### Study Design

We performed a cross-sectional study of the prevalence and determinants of noncommunicable respiratory disease among adults living in Chikhwawa District, Malawi.

### Setting

Chikhwawa is ∼50 km from the nearest city, Blantyre, on the southern Shire River Valley, and it consists primarily of subsistence farmers living in rural village communities. The Malawi College of Medicine Research Ethics Committee (Ethics Committee reference no. P.11/12/1308) and the Liverpool School of Tropical Medicine Research Ethics Committee (Ethics Committee reference no. 12.40) approved the CAPS trial protocol that includes this work, a summary of which was published by *The Lancet* (12).

### Participants

Following community engagement events that included village leaders and other community representatives, a list of all the adults living in each of the 50 villages participating in CAPS in Chikhwawa was obtained from local community health workers known as Health Surveillance Assistants. These lists were collated and used by an independent statistician at the BOLD (Burden of Obstructive Lung Disease) center in London to obtain a population-representative sample of adults >18 years of age with stratification by age and sex. All potential participants sampled in this way were then individually invited to participate with written informed consent (or witnesses thumbprint for those unable to read and write) obtained from those who agreed. People who were acutely unwell, not permanent residents, or pregnant were excluded.

### Procedures

Fieldworkers who had undergone study-specific training and met the required quality standards did home visits according to standardized operating procedures. With the exception of the air pollution monitoring procedures that are not part of the BOLD study protocol, all procedures were conducted in accordance with the BOLD study protocol, which has been described previously (13). Minimal demographic information was collected from participants who declined to participate in the full study. Fieldworkers administered BOLD study questionnaires in the local language, Chichewa. Height and weight were measured using a portable stadiometer and scales. All eligible participants were asked to do before and after bronchodilator spirometry, which BOLD center–certified fieldworkers performed to European Respiratory Society/American Thoracic Society guidelines using the ndd EasyOne spirometer (ndd Medical Technologies) (14). Up to three repeat visits were arranged to achieve the required spirometry quality standards. Spirometry data were sent electronically to the BOLD center for quality control.

Personal exposures to particulate matter <2.5 μm in aerodynamic diameter (PM_2.5_) and carbon monoxide (CO) were measured continuously for 48 hours using the indoor air pollution (IAP) 5000 series monitor (Aprovecho Research Center). The IAP 5000 sampled air from the breathing zone using a short tube and logged continuous PM_2.5_ and CO using a light-scattering photometer and an electrochemical cell CO sensor, respectively. All monitors were calibrated at the Aprovecho Research Center prior to use in the study. Monitors were worn in small backpacks apart from during sleep, when they were kept beside the sleeping mat or bed.

### Variables

Clinical outcomes were presence or absence of specific symptoms as assessed by a questionnaire. The questions (outcomes) asked were as follows: Do you usually have a cough when you don’t have a cold (cough outcome)? Do you usually bring up phlegm from your chest (phlegm outcome)? Have you had wheezing/whistling in your chest at any point in the last 12 months, in the absence of a cold (wheeze outcome)? Do you have shortness of breath when hurrying on the level or walking up a slight hill (dyspnea outcome)? And have your breathing problems interfered with your daily activities (functional limitation outcome)? A composite variable for any symptoms was created by defining as positive if an individual reported any of the above symptoms (any symptoms outcome).

Continuous FEV_1_ and FVC spirometry values were used in the primary analysis. Spirometric obstruction and restriction were defined according to the NHANES III white reference range lower limits of normal (15).

Exposures of interest included personal exposure to PM_2.5_ or CO as measured by the personal monitoring device, and two exposures assessed by questionnaire: smoking status and previous episode of tuberculosis (TB). A questionnaire-assessed variable asking for any biomass exposure was considered, but as most (>99%) indicated yes, it was not included in any modeling.

Raw PM_2.5_ and CO exposures were corrected for background levels using calibration values for each monitoring period. In cases where calibration data were missing or corrupted (<5%), aggregated mean calibration values were used. Observations where <2,000 minutes of time were recorded were excluded, as were monitoring periods affected by device malfunction. Both PM_2.5_ and CO were log_10_ transformed for presentation and inclusion in models due to large positive skew.

Potential confounders/effect modifiers included were body mass index (BMI) and/or height (cm) and weight (kg) variables, as well as age, years of education, and sex.

### Study Size

We initially invited 2,000 people to participate but increased this to 3,000 to achieve the required sample size. Participants were stratified into two age groups: 18–39 years and ≥40 years. We estimated that, after allowing for unequal age and sex distributions, refusals, and inability to provide spirometry measurements of acceptable quality, a sample of just 300 participants in any one sex/age stratum (1,200 total) would provide an estimate of chronic airflow limitation prevalence in this stratum with a precision (95% confidence interval [CI]) of ±3.3% to ±5.0% assuming a prevalence of 10–25%.

### Statistical Analyses

Univariate analysis was completed using descriptive statistics to explore the characteristics of the study population. Descriptive analysis is presented for clarity using categorical versions of BMI (underweight, normal, overweight, or obese) and categorical versions of age; however, age, weight, and height were entered into models as continuous variables. Participants who completed the study in full or in part were assessed for selection bias using the χ^2^ and Student’s *t* tests. Multivariable logistic regression was used to estimate the strength of the association between measured exposure variables and dichotomous clinical outcomes, adjusting for potential confounders. All models were adjusted for age, sex, weight, and height. Linear multivariable regression was used to estimate the association between exposures and continuous lung function values (FEV_1_, FVC, and FEV_1_/FVC). Secondary exploratory trial efficacy analyses were by intention to treat. Statistical significance was nominally set at α = 0.05. Stata version 14.2 and R version 3.4 statistical software was used for data analysis (Stata statistical software: R.14; StataCorp, LLC).

### Role of the Funding Source

The funders had no role in the study design, data collection, analysis, interpretation, or writing of the report. The corresponding author had full access to all the study data and had final responsibility for the decision to submit for publication.

## Results

**Figure 1.**
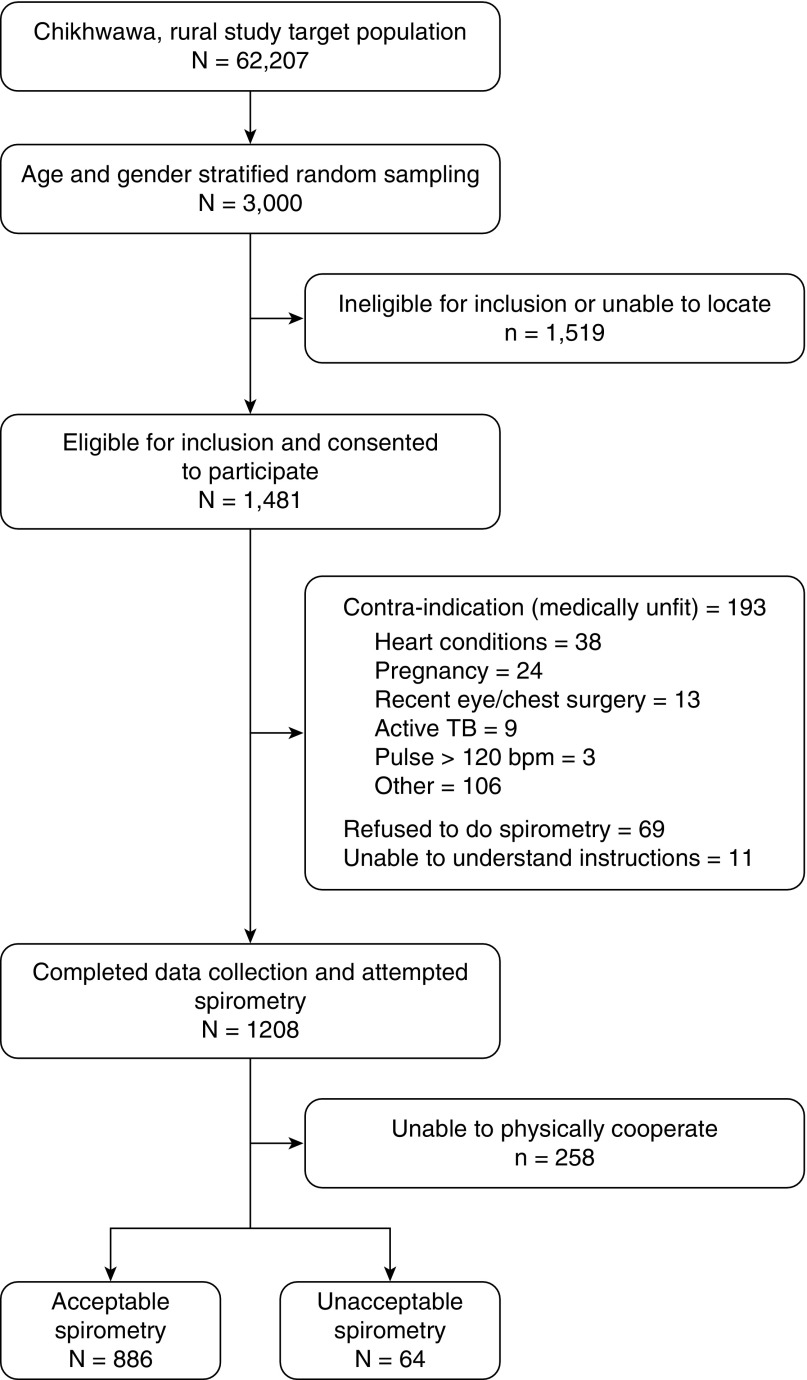
Participant recruitment flow diagram. TB = tuberculosis.

Between August 2014 and July 2015, we attempted to contact the 3,000 adults sampled to invite them to participate, of whom 1,481 (49.4%) consented and completed BOLD study questionnaires. Of these, 950 (64.6%) went on to do spirometry; the remaining 520 (35.3%) were unable to do spirometry because they could not physically cooperate with the procedure (*n* = 258; 48.9%), had a fieldworker-determined contraindication (*n* = 193; 37%), or refused (*n* = 69; 13.3%). Of the 1,481 participants, 1,144 (77.2%) underwent personal air pollution exposure monitoring. There were 424 (28.6%) participants from CAPS intervention or control households (Figure 1).

The mean age (SD) of participants was 43.8 (17.8) years and 57% were female (Table 1). Just more than half of the participants had been educated only to primary school level, with a third having had no formal school education. The use of biomass fuels for cooking was almost universal (99.8%).

**Table 1.** Demographic and Clinical Characteristics (*N* = 1,481)

Characteristic	Level	*n* (*%*) or Mean (SD)
Age group, yr	<39	686 (46.32)
	40–49	259 (17.49)
	50–59	216 (14.58)
	60–69	160 (10.80)
	>70	160 (10.80)
		
Sex	Male	637 (43.01)
	Female	844 (56.99)
		
Education	None	485 (32.79)
	Primary	758 (51.25)
	Middle	205 (13.86)
	High school or college	31 (2.10)
	Missing	2 (0.0)
	Years of education, mean (SD)	4.20 (4.09)
	Years of education if any, mean (SD)	6.31 (3.44)
		
Smoking	Never smoker	1,152 (77.8)
	Current or ever smoker	328 (22.2)
		
Pack-years of smoking	0	1,152 (77.8)
	Up to 10 pack-years	263 (17.8)
	>10 pack-years	63 (4.3)
	Missing	3 (0.0)
		
BMI group, kg/m^2^	Underweight (BMI, <18.5)	188 (14.4)
	Normal (BMI, 18.5–25)	945 (72.5)
	Overweight (BMI, 25–30)	130 (10.0)
	Obese (BMI, >30)	40 (3.1)
	Missing	178 (12.0)
		
Previous TB	No	1,434 (92.3)
	Yes	47 (3.2)
		
Symptoms	Cough (Do you usually cough when you don’t have a cold?)	165 (11.1)
	Sputum (Do you usually bring up phlegm from your chest?)	38 (2.6)
	Wheeze (Have you had wheezing/whistling in your chest at any point in past 12 months in the absence of a cold?)	23 (1.6)
	MRC dyspnea II (Do you have shortness of breath when hurrying on the level or walking up a slight hill?)	23 (1.6)
	Any respiratory symptoms (Any of cough, sputum, wheeze without cold, exertional breathlessness as above?)	201 (13.6)
	Functional limitation (Have breathing problems interfered with your usual daily activities?)	43 (2.9)

*Definition of abbreviations*: BMI = body mass index; MRC = Medical Research Council; TB = tuberculosis.

Data are *n* (%) unless otherwise indicated.

**Figure 2.**
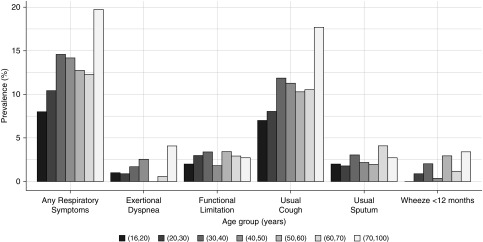
Age-stratified prevalence of respiratory symptoms.

One or more chronic respiratory symptom was reported by 201 (13.6%; 95% CI, 11.9–15.4) participants (Table 1 and Figure 2). Respiratory symptoms were more commonly reported with increasing age. Regular cough was reported by 11.1% (95% CI, 9.6–12.8) while 2.6% (95% CI, 1.9–3.5) reported usually coughing up phlegm. Breathlessness and wheeze were less commonly reported: 1.6% (95% CI, 1.0–2.3) and 1.6% (95% CI, 1.0–2.3), respectively. Respiratory symptoms that limited functional ability were reported by 2.9% (95% CI, 2.2–3.9). A previous diagnosis of TB was reported by 3.2% (95% CI, 2.4–4.2), which was more common with increasing age. Current or former smoking was reported by 22.1% (95% CI, 20.1–24.3), although only 4.3% had a >10 pack-year history. Many participants (14.4%) had a low BMI.

Of the 950 participants who did spirometry, 886 (93.2%) achieved BOLD study quality standards and were included in the analyses. Factors associated with declining or not completing spirometry to European Respiratory Society/American Thoracic Society standards were older mean age (48 vs. 39 yr; *P* < 0.001), being female (65% vs. 51%; *P* < 0.001), lower mean years of education (2 vs. 5 yr; *P* < 0.001), and lower mean BMI (20.7 vs. 21.3; *P* < 0.001). As shown in Table E1 in the online supplement, participants who completed spirometry were less likely to have cough, wheeze, and dyspnea compared with those who did not complete spirometry and were slightly more likely to have phlegm and functional limitation, although none of these differences was statistically significant. Spirometric obstruction and restriction were present in 8.7% (95% CI, 7.0–10.7) and 34.8% (95% CI, 31.7–38.0) of the 886 participants who met the required quality standards, respectively.

**Figure 3.**
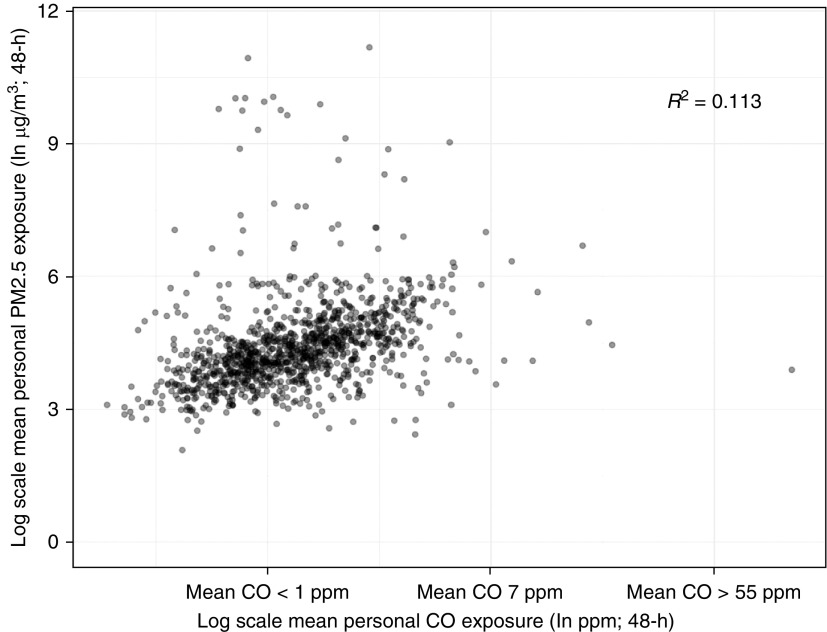
Scatter plot between personal exposure to particulate matter <2.5 μm in aerodynamic diameter (PM_2.5_) and carbon monoxide (CO).

Of the 1,144 participants (mean age [SD], 43.9 [17.9] yr; 57% female) who underwent personal exposure monitoring, 1,117 (97.6%) had valid exposure monitoring records. The 48-hour median personal PM_2.5_ and CO exposures were 71.0 μg/m^3^ (interquartile range [IQR], 44.6–119.2) and 1.23 ppm (IQR, 0.79–1.93), respectively. There was weak correlation between these two air pollution exposure measures (Figure 3).

In logistic multivariable analysis, smoking (odds ratio [OR], 1.56; 95% CI, 1.01–2.41) and previous TB (OR, 2.81; 95% CI, 1.19–6.08) were associated with cough (Table 2 and Table E1). In continuous multivariable analysis, both FEV_1_ and FVC had a negative association with increasing age and were higher for men compared with women (Table 3). Smoking (coefficient estimate, −0.09; 95% CI, −0.16 to −0.01) and previous TB (coefficient estimate, −0.46; 95% CI, −0.64 to −0.28) were associated with FEV_1_, and previous TB was associated with FVC (coefficient estimate, −0.35; 95% CI, −0.56 to −0.15). There was no association between personal exposure to PM_2.5_ and any of the demographic and clinical characteristics or spirometric indices (Tables 4 and 5). The only statistically significant association was between exposure to CO and reporting “any chronic respiratory symptoms” (OR, 1.46; 95% CI, 1.04–2.05). There were no statistically significant associations between personal exposure to CO and any other demographic and clinical characteristics or to any spirometric indices. There were 424 (227 intervention; 197 control) participants in the CAPS intention-to-treat population; however, not all of them had complete spirometry (133 without) or exposure measures (87 without). There were no differences in respiratory symptoms, spirometric indices, or exposure to CO or PM_2.5_ between the intervention and control groups (Table 6).

**Table 2.** Odds Ratios (95% Confidence Interval) for Chronic Respiratory Symptom Outcomes Estimated by Multivariable Logistic Regression

Variable	Cough	Phlegm*	Wheeze*	Dyspnea*	Functional Limitation	Any Symptoms
Age, yr	1.01 (1.00–1.02)	1.00 (0.97–1.02)	1.02 (0.99–1.05)	1.01 (0.98–1.04)	1.00 (0.95–1.02)	1.00 (0.97–1.02)
Male	Ref	Ref	Ref	Ref	Ref	Ref
Female	0.78 (0.49–1.25)	1.02 (0.42–2.51)	0.97 (0.30–3.28)	3.08 (0.88–11.65)	1.17 (0.28–2.37)	1.08 (0.70–1.67)
Never smoked	Ref	Ref	Ref	Ref	Ref	Ref
Ever smoked	1.56 (1.01–2.41)	1.37 (0.58–3.15)	0.77 (0.20–2.47)	1.85 (0.51–6.07)	0.65 (0.18–1.93)	1.59 (1.05–2.39)
Previous TB: no	Ref	—	—	—	Ref	Ref
Previous TB: yes	2.81 (1.19–6.08)	—	—	—	2.64 (0.40–9.95)	2.50 (1.04–15.58)
Years of education	0.97 (0.92–1.02)	0.90 (0.81–1.00)	0.99 (0.86–1.13)	0.96 (0.83–1.10)	1.06 (0.96–1.16)	0.98 (0.93–1.03)

*Definition of abbreviations*: Ref = reference; TB = tuberculosis.

All models were also adjusted for weight (kg) and height (cm); total *n* = 1,303 owing to missing weight data.

* Only one person had both TB and wheeze, one person had both TB and phlegm, and one person had both TB and dyspnea; TB was excluded from these models.

**Table 3.** Coefficient Estimates (95% Confidence Interval) for Continuous Spirometry Outcomes FEV_1_, FVC, and FEV_1_/FVC Ratio (*n* = 886)

Variable	FEV_1_	FVC	FEV_1_/FVC
Age, yr	−0.02 (−0.02 to −0.02)	−0.01 (−0.01 to −0.01)	−0.28 (−0.31 to −0.24)
Male	Ref	Ref	Ref
Female	−0.53 (−0.60 to −0.45)	−0.70 (−0.78 to −0.62)	1.37 (0.18 to 2.56)
Never smoked	Ref	Ref	Ref
Ever smoked	−0.09 (−0.16 to −0.01)	−0.05 (−0.14 to 0.04)	−1.76 (−2.99 to −0.53)
Previous TB: no	Ref	Ref	Ref
Previous TB: yes	−0.46 (−0.64 to −0.28)	−0.36 (−0.56 to −0.15)	−7.83 (−10.74 to −4.91)
Years of education	0 (0 to 0.01)	0 (−0.01 to 0.01)	0.18 (0.05 to 0.3)

*Definition of abbreviations*: Ref = reference; TB = tuberculosis.

All models were also adjusted for weight (kg) and height (cm).

**Table 4.** Odds Ratios (95% Confidence Interval) for Symptom Outcomes Estimated by Multivariable Logistic Regression in Participants with Exposure Measurements (*n* = 985)

	Cough	Phlegm*	Wheeze*	Dyspnea*	Functional Limitation	Any Symptoms
Ever smoked (ref: never smoked)	1.72 (1.02–2.91)	0.99 (0.37–2.53)	0.35 (0.02–2.32)	2.62 (0.56–11.24)	0.78 (0.20–2.47)	1.67 (1.02–2.71)
Previous TB (ref: no previous TB)	2.87 (1.07–6.87)	—	—	—	3.00 (0.5–11.74)	2.47 (0.91–6.07)
CO (log_10_ ppm)	1.29 (0.93–1.77)	1.50 (0.83–2.54)	2.12 (0.96–4.16)	1.27 (0.48–2.88)	1.45 (0.81–2.43)	1.46 (1.04–2.05)
PM_2.5_ log_10_ μg/m^3^)	1.02 (0.95–1.13)	0.96 (0.88–1.11)	1.00 (0.87–1.38)	1.11 (0.89–1.67)	0.99 (0.90–1.16)	1.02 (0.95–1.11)

*Definition of abbreviations*: CO = carbon monoxide; PM_2.5_ = particulate matter <2.5 μm in aerodynamic diameter; ref = reference; TB = tuberculosis.

All models were adjusted for weight (kg), height (cm), age (yr), sex (male, female), and years of formal education.

* Only one person had both TB and wheeze, one person had both TB and phlegm, and one person had both TB and dyspnea; TB was excluded from these models.

**Table 5.** Coefficient Estimates (95% Confidence Interval) for Continuous Spirometry Outcomes FEV_1_, FVC, and FEV_1_/FVC Ratio in Participants with Personal Air Pollution Exposure Measurements (*n* = 886)

Variable	FEV_1_	FVC	FEV_1_/FVC
Age, yr	−0.02 (−0.02 to −0.01)	−0.01 (−0.02 to −0.01)	−0.28 (−0.35 to −0.20)
Male	Ref	Ref	Ref
Female	−0.58 (−0.61 to −0.44)	−0.70 (−0.79 to −0.60)	1.25 (−0.09 to 2.56)
Never smoked	Ref	Ref	Ref
Ever smoked	−0.1 (−0.19 to −0.02)	−0.07 (−0.16 to 0.03)	−1.83 (−3.20 to −0.45)
Previous TB: no	Ref	Ref	Ref
Previous TB: yes	−0.32 (−0.52 to −0.11)	−0.26 (−0.49 to −0.02)	−6.16 (−9.48 to −2.85)
Years of education	0 (0 to 0.01)	0 (−0.01 to 0.01)	0.15 (0.01 to 0.29)
CO (log_10_ ppm)	0.01 (−0.04 to 0.06)	0.01 (−0.04 to 0.07)	0.13 (−0.68 to 0.94)
PM_2.5_ (log_10_ μg/m^3^)	0 (−0.02 to 0.01)	0 (−0.01 to 0.01)	−0.11 (−0.29 to 0.08)

For definition of abbreviations, *see* Table 4.

All models were also adjusted for weight (kg) and height (cm).

**Table 6.** CAPS Intention-to-Treat Secondary Trial Analyses (*n* = 424)

Outcome	Intervention (*n* = *227*)	Control (*n* = *197*)	Intervention vs. Control Coefficient Estimate (95% CI)	*P* Value
Symptoms, *n* (%)	22 (9.7)	26 (13.2)	0.90 (0.45 to 1.82)*	0.87
FEV_1_, median (IQR)	2.81 (2.39 to 3.26)	2.77 (2.40 to 3.10)	0.08 (−0.06 to 0.22)	0.26
FVC, median (IQR)	3.37 (2.88 to 3.91)	3.31 (2.83 to 3.86)	0.04 (−0.13 to 0.21)	0.62
Mean CO, median (IQR)	1.13 (0.79 to 1.90)	1.28 (0.82 to 1.79)	0.67 (−0.60 to 1.96)	0.30
Mean PM_2.5_, median (IQR)	67.90 (44.72 to 112.95)	64.47 (40.73 to 101.80)	−931.6 (−2,073.6 to 209.7)	0.11

*Definition of abbreviations*: CAPS = Cooking and Pneumonia Study; CI = confidence interval; CO = carbon monoxide; IQR = interquartile range; PM_2.5_ = particulate matter <2.5 μm in diameter.

Mean exposure per individual is calculated, and the median (IQR) of those values is reported.

* Odds ratio (95% CI).

## Discussion

The main findings of this cross-sectional study of the burden and determinants of noncommunicable respiratory disease in adults living in Chikhwawa, rural Malawi, were that: 13.6% of participants had chronic respiratory symptoms (mainly cough); >40% had abnormal spirometry (mainly spirometric restriction); day-to-day air pollution exposures were approximately three times the WHO upper safety limit; and there was no association between CAPS trial arm and any of the secondary trial outcomes in the subset of adults included both in this study and the trial.

The finding of a low prevalence of spirometric obstruction in this setting—where highly polluting fuels are almost universally used for household energy needs and where exposure to household air pollution is high—is surprising given that household air pollution–associated COPD has been suggested to be a major global health problem and as such would be expected to be highly prevalent in our study setting (16–19). This finding is consistent with an emerging body of evidence challenging the dogma that exposure to household air pollution is a major cause of COPD, including a recent pooled analysis of BOLD study data from low-, middle-, and high-income countries (20). This analysis found no association between spirometric obstruction and self-reported use of solid fuels for cooking or heating. This is, however, an area of controversy, with investigators disagreeing about the interpretation of the available data (21, 22).

Many of the studies conducted to date looking at the association between COPD and exposure to household air pollution have had important methodological limitations, including case definition and exposure assessments. So far, studies of the long-term effects of air pollution have had to use a self-reported history of exposure with all the limitations that this may imply. To improve exposure assessment, we included 48 hours of personal air pollution exposure measurements in study participants in addition to questionnaire-based exposure assessments. Although this approach and the particular devices used have their limitations, by doing this we were able to deliver the first study of the burden of noncommunicable lung disease anywhere in the world to incorporate BOLD study methodology and measurements of personal air pollution exposure and to do so in almost 1,000 participants.

Although personal air pollution exposure levels were undoubtedly high and at levels at which adverse health effects would be expected, and although widely considered a risk factor for noncommunicable respiratory diseases and COPD in particular, the only respiratory outcome associated with measured exposure to PM_2.5_ or CO was “any chronic respiratory symptoms” with increased CO exposure. Interestingly, whether questionnaire-based or directly measured personal air pollution exposure assessments were used, there was no significant association between air pollution exposure and an increased risk of spirometric abnormalities. However, we acknowledge that 48-hour measurements of air pollutants may not be an adequate surrogate for cumulative exposure to household air pollution that has been associated, albeit by self-report, with COPD. Our observations, taken together with the findings of other recent studies, bring into question the extent to which household air pollution and other sources of air pollution play in the development of abnormal lung function in rural African settings similar to this one in rural Malawi. It is plausible that the levels of personal exposure to air pollution seen are not high enough to accelerate lung function decline and the development of airflow obstruction in the way that tobacco smoke does; a prospective cohort study of the rate of decline in lung function in relationship to air pollution exposures is needed in adults in sub-Saharan Africa to explore this further.

This study benefited from being conducted at the same time and in the same villages as CAPS, which presented the opportunity to look for an effect of the CAPS intervention on respiratory symptoms, spirometric indices, and personal air pollution exposures in a subsample of adults. Consistent with the main trial findings of no effect of the intervention on pneumonia in children <5 years of age (5), we found no evidence that the intervention was associated with beneficial effects on any of these trial secondary outcome measures among adults. However, these analyses were exploratory secondary analyses limited by a relatively small number of participants and therefore statistical power to detect effects and, although sufficient to see an effect on symptoms and air pollution exposures, there was limited time between intervention and outcome assessment for potential effects on spirometric indices to be seen. Other possible explanations for the lack of effect of the CAPS intervention on these outcomes include insufficient levels of intervention adoption, insufficient reductions in emissions and exposures, and other sources of air pollution exposure overwhelming any potential effect of the intervention (5).

A notable observation of this study was that 35% of participants had spirometric restriction when benchmarked against NHANES III white reference range values. We consider the approach we have taken of benchmarking against the NHANES III white reference ranges as the best we can do at this time while accepting that this and all other currently available alternatives are not ideal. That includes locally derived reference ranges that might be helpful in defining what is ‘usual’ lung function in asymptomatic nonsmoking Malawian adults but that may be far from “optimal potential normal lung function.” Because there is evidence that the prognostic significance of spirometric restriction holds irrespective of racial/ethnic group when benchmarked in this way (23, 24), the finding of such a high burden of spirometric restriction in the rural Malawian population, and elsewhere in sub-Saharan Africa (25, 26), is of considerable concern; observational cohort studies are needed to understand the clinical characteristics and prognostic significance of these findings. The underlying drivers of spirometric restriction in sub-Saharan African populations are not yet understood, but we hypothesize that these are primarily environmental insults experienced in early life (e.g., malnutrition, infections and air pollution exposures before conception, *in utero*, and during childhood) such that adulthood is reached without maximal potential lung function having been achieved. Cross-sectional studies of the burden and determinants of noncommunicable lung disease in children in sub-Saharan Africa are needed to explore whether the same patterns of abnormality are seen in early life and, if so, studies even earlier in the life course to identify potential windows of opportunity to intervene to maximize lung health.

Strengths of this study include that it was conducted in a highly challenging research setting in one of the world’s poorest rural communities as part of the CAPS protocol; it is the first of the global BOLD studies to be conducted in a rural sub-Saharan African setting; and it is also the first BOLD study to incorporate personal air pollution exposure measurements and to do so at scale. Limitations include questionnaire assessments for most variables with potential for recall bias; the potential bias (e.g., underestimation of the burden of spirometric abnormalities) caused by participants who did not do spirometry, although the quality of those that did spirometry was generally high; and air pollution exposure assessments that provided only a 48-hour snapshot of exposure and were based on a light-scattering method alone for PM_2.5_.

In conclusion, we found that exposures to air pollution among Malawian adults living in communities participating in CAPS were at levels well beyond those considered safe by the WHO. In keeping with the primary outcome of the CAPS trial, we found no effect of the intervention on any of the secondary trial outcomes (*i.e.*, respiratory symptoms, spirometric indices, or air pollution exposures) in the subsample of adults participating in both this study and the trial. The prevalence of chronic respiratory symptoms and abnormal spirometry suggests that there may be an important burden of noncommunicable respiratory disease in these communities. The characteristics of noncommunicable respiratory disease in sub-Saharan Africa may be different to those previously expected, with more spirometric restriction and less obstruction (and household air pollution–associated COPD) than has been thought to exist. There is a need to explore other plausible explanations for the poor lung function observed in these and other low- and middle-income country populations, including further exploration of the role of TB, recurrent pneumonia, and nutrition. Clinically effective and cost-effective approaches for the prevention and control of noncommunicable respiratory diseases are very much needed in sub-Saharan Africa.

## Supplementary Material

Supplements

Author disclosures
