# Supplementary material for: Noncommunicable Respiratory Disease and Air Pollution Exposure in Malawi (CAPS). A Cross-Sectional Study
Source: Am J Respir Crit Care Med. 2019 Mar 1;199(5):613–21. doi: 10.1164/rccm.201805-0936OC (PMC6396863; doi:10.1164/rccm.201805-0936OC)
Supplement: Supplements [file rccm.201805-0936OC.html]

Noncommunicable Respiratory Disease and Air Pollution Exposure in Malawi (CAPS). A Cross-Sectional Study | American Journal of Respiratory and Critical Care Medicine

- nightingale\_data\_supplement.pdf (126 KB)
- disclosures.pdf (169 KB)
