# Supplementary material for: Noncommunicable Respiratory Disease and Air Pollution Exposure in Malawi (CAPS). A Cross-Sectional Study
Source: Am J Respir Crit Care Med. 2019 Mar 1;199(5):613–21. doi: 10.1164/rccm.201805-0936OC (PMC6396863; doi:10.1164/rccm.201805-0936OC)
Supplement: Supplements [file rccm.201805-0936OC_nightingale_data_supplement.pdf]

Non-Communicable Respiratory Disease and Air Pollution Exposure in Malawi (CAPS): A Cross-Sectional Study

Rebecca Nightingale (MRes), Maia Lesosky (PhD), Graham Flitz (MS),
Sarah J. Rylance (Mres), Jamilah Meghji (MPH), Peter Burney (MD), John Balmes (MD),
and Kevin Mortimer (PhD)

ONLINE DATA SUPPLEMENT

Online Data Supplement

Table E1: OR (95% CI) for chronic respiratory symptom outcomes estimated by multivariable logistic regression as shown in Table 2 but with the addition of a variable for completing or not completing spirometry

Variable	Cough	Phlegm**	Wheeze**	Dyspnoea**	Functional limitation	Any symptoms
**Age (years)**	1·01 (0·99, 1·02)	1·00 (0·99, 1·02)	1·02 (0·99, 1·05)	1·01 (0·98, 1·04)	1·00 (0·97, 1·02)	1·01 (1.00, 1.02)
**Male**	ref	ref	ref	ref	ref	ref
**Female**	0·77 (0·48, 1·25)	1·02 (0·42, 2·51)	0·97 (0·30, 3·28)	3·08 (0·88, 11·65)	1·18 (0·47, 3·11)	1·07 (0.69, 1.65)
**Never smoked**	ref	ref	ref	ref	ref	ref
**Ever smoked**	1·57 (1·02, 2·41)	1·37 (0·58, 3·15)	0·77 (0·20, 2·47)	1·85 (0·51, 6·07)	0.65 (0.18, 1·93)	1.59 (1.05, 2.38)
**Previous TB:**						
**No**	ref	-	-	-	ref	ref
**Yes**	2·84 (1·21, 6·08)				2·64 (0.40, 9·94)	2·52 (1.05, 5.62)
**Years education**	0·97 (0·92, 1·02)	0·90 (0·81, 1.00)	0·99 (0·86, 1·13)	0·96 (0·83, 1·10)	1.06 (0·96, 1·16)	0.98 (0·93, 1·03)
**Spiro complete**						
**No**	ref	ref	ref	ref	ref	ref
**Yes**	0.73 (0.5, 1.06)	1.06 (0.51, 2.35)	0.74 (0.28, 2.02)	0.80 (0.30, 2.28)	1.11 (0.53, 2.52)	0.82 (0.58, 1.17)

All models also adjusted for weight (kg), height (cm);
** Only one person had both TB and wheeze or TB and phlegm or TB and dyspnoea, TB was excluded from these models.
